# Intracellular amastigotes in fine‐needle aspiration cytology of cutaneous leishmaniasis

**DOI:** 10.1002/ccr3.7377

**Published:** 2023-05-22

**Authors:** Prajwal Pudasaini, Sagar GC, Sushil Paudel

**Affiliations:** ^1^ Civil Service Hospital Kathmandu Nepal

**Keywords:** cutaneous leishmaniasis, FNAC, intracellular amastigotes

## Abstract

We report a case of 45‐year‐old farmer who presented with solitary crateriform non healing ulcer with crust over left dorsal hand. FNAC of lesion showed intracellular round to oval amastigotes within macrophage on giemsa stain. This simple diagnostic method could be utilized as a diagnostic tool in resource poor setting.

## 1 QUESTION

**FIGURE 1:**
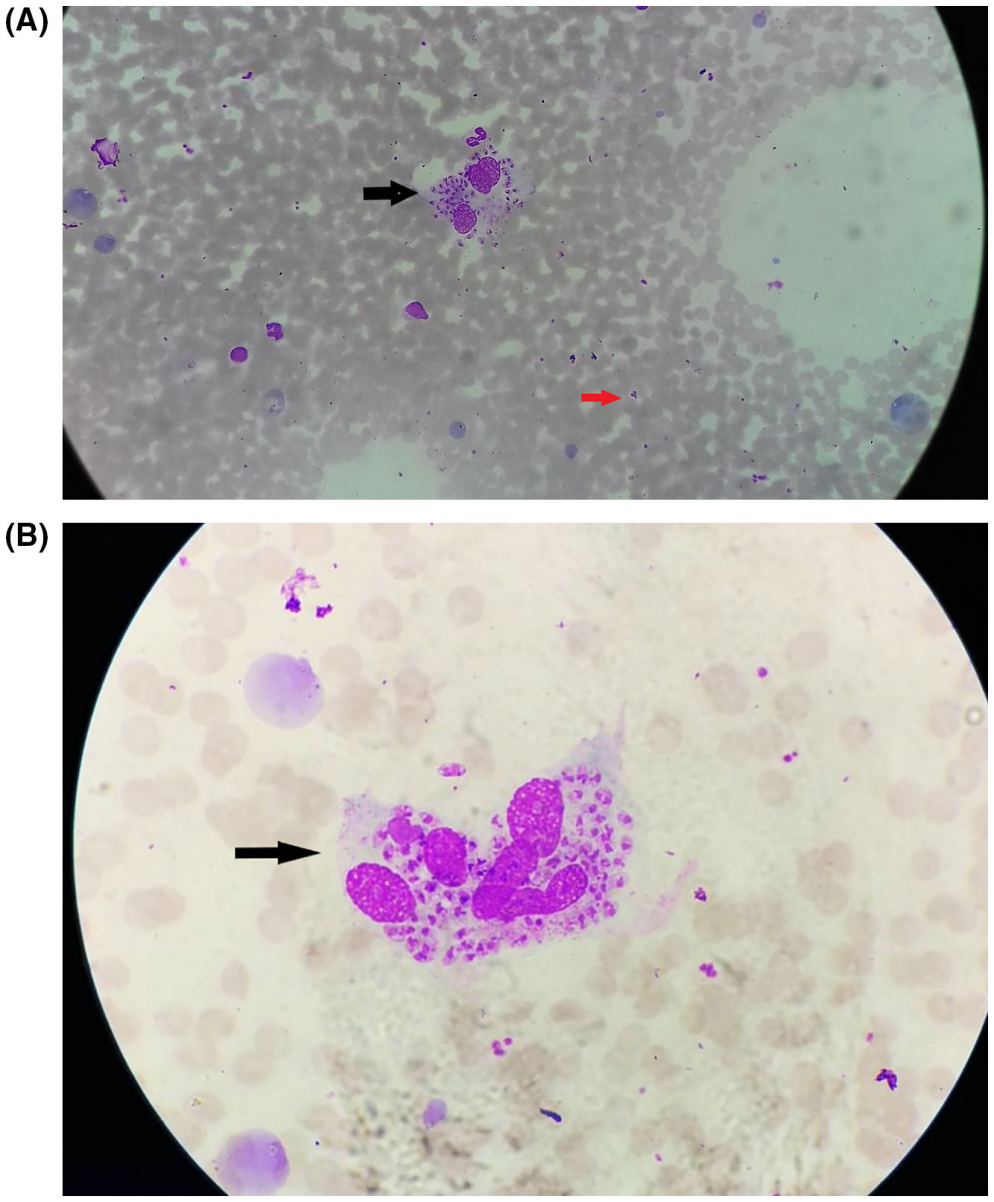
(A) Multiple intracellular amastigotes of leishmania species within macrophage as seen on Giemsa stain in FNAC (black arrow) and extracellular amastigote in same smear (red arrow). (B) Intracellular amastigotes seen within macrophage, with eccentric nuclei and kinetoplast.

A 45‐year‐old farmer from rural village of Syangja, Nepal, with frequent farm work, presented with lesion over left dorsal hand for 4 months. Initially, solitary acneiform lesion was present that progressed over period of 10 days to form crateriform non healing ulcer with central crust. FNAC showed round to oval intracellular amastigotes within the macrophage on giemsa stain. Amastigote showed eccentric nucleus, and some of them had kinetoplast (Figure 1A,B). What do these findings suggest and what to do next?

## 2 DISCUSSION

Leishmaniasis is a protozoal infection caused by leishmania species transmitted via bite of infected female Phlebotomus sandfly on exposed site. There are varied clinical phenotypic presentation of leishmaniasis which ranges from cutaneous to multisystemic effects.^1^ Cutaneous leishmaniasis caused by leishmania species has been on rise in recent times in rural parts of Nepal.^2^ Due to similar other clinical mimics, its diagnosis is often confused with other endemic disease such as lupus vulgaris. Hence, a simple yet diagnostic technique such as FNAC will be helpful for diagnosis through identification of intracellular amastigotes in dermal macrophages of human hosts in resource poor setting of the low‐income countries.^3^ Corroboration of clinical features with parasitological confirmation will defer sophisticated tests such as polymerase chain reaction (PCR) for identification of leishmanial DNA in rural parts of Nepal. Hence, a simple and easily available confirmatory diagnostic test such as FNAC could be utilized in resource poor setting. Treatment includes intravascular or intramuscular injection of sodium stibogluconate 20 mg/kg for 15–21 days or until clinical or parasitological resolution.

## AUTHOR CONTRIBUTIONS

**Prajwal Pudasaini:** Conceptualization; data curation; investigation; supervision; writing – original draft; writing – review and editing. **Sagar GC:** Conceptualization; investigation; writing – original draft; writing – review and editing. **Sushil Paudel:** Conceptualization; formal analysis; methodology; resources; supervision; writing – original draft; writing – review and editing.

## FUNDING INFORMATION

None.

## CONFLICT OF INTEREST STATEMENT

None.

## CONSENT

Written informed consent was obtained from the patient to publish this report in accordance with the journal's patient consent policy.

## Data Availability

The data that support the findings of this study are openly available in clinical case reports.

## References

[1] Mokni M . Leishmanioses cutanées. Ann Dermatol Venereol. 2019;146(3):232‐246. 30879803 10.1016/j.annder.2019.02.002

[2] Pandey K , Bastola A , Haiyan G , Pyakurel UR , Pandey BD , Dumre SP . Emergence of cutaneous leishmaniasis in Nepal. Trop Med Health. 2021;49(1):1‐9. 34503578 10.1186/s41182-021-00359-3 PMC8428101

[3] Pudasaini P , Pudasaini P . Cutaneous leishmaniasis: a case report of a diagnostic dilemma. Clin Case Rep. 2022;10:e05428. doi:10.1002/ccr3.5428 35198202 PMC8841021

